# Combing Transcriptomes for Secrets of Deep-Sea Survival: Environmental Diversity Drives Patterns of Protein Evolution

**DOI:** 10.1093/icb/icz063

**Published:** 2019-05-29

**Authors:** J R Winnikoff, W R Francis, E V Thuesen, S H D Haddock

**Affiliations:** 1 Monterey Bay Aquarium Research Institute, 7700 Sandholdt Rd., Moss Landing, CA 95039, USA; 2 Ecology and Evolutionary Biology, University of California at Santa Cruz, 130 McAllister Way, Santa Cruz, CA 95060, USA; 3 Department of Biology, University of Southern Denmark, Campusvej 55, 5230 Odense, Denmark; 4 The Evergreen State College, Laboratory I, Olympia, WA 98505, USA

## Abstract

Ctenophores, also known as comb jellies, live across extremely broad ranges of temperature and hydrostatic pressure in the ocean. Because various ctenophore lineages adapted independently to similar environmental conditions, Phylum Ctenophora is an ideal system for the study of protein adaptation to extreme environments in a comparative framework. We present such a study here, using a phylogenetically-informed method to compare sequences of four essential metabolic enzymes across gradients of habitat depth and temperature. This method predicts convergent adaptation to these environmental parameters at the amino acid level, providing a novel view of protein adaptation to extreme environments and demonstrating the power and relevance of phylogenetic comparison applied to multi-species transcriptomic datasets from early-diverging metazoa. Across all four enzymes analyzed, 46 amino acid sites were associated with depth-adaptation, 59 with temperature-adaptation, and 56 with both. Sites predicted to be depth- and temperature-adaptive occurred consistently near Rossmann fold cofactor binding motifs and disproportionately in solvent-exposed regions of the protein. These results suggest that the hydrophobic effect and ligand binding may mediate efficient enzyme function at different hydrostatic pressures and temperatures. Using predicted adaptive site maps, such mechanistic hypotheses can now be tested via mutagenesis.

## Introduction

Organisms like ctenophores, cnidarians, and sponges are notable for having colonized a vast diversity of habitats. Ctenophores, for instance, can be found from the equator to polar seas and from sea level to over 7000 m deep. Since ctenophores tend to lack physical protection for their cells, much of their prodigious environmental adaptation is biochemical. As such, it implicates changes in certain vital proteins, such as metabolic “housekeeping” enzymes, whose performance is sensitive to the environment. These changes should in turn be traceable to organisms’ transcriptomes. Despite the growing abundance of transcriptomes available for early-diverging taxa from a wide variety of environments, techniques for correlating protein-coding sites to phenotypes in multi-species datasets remain underdeveloped.

**Fig. 1:**
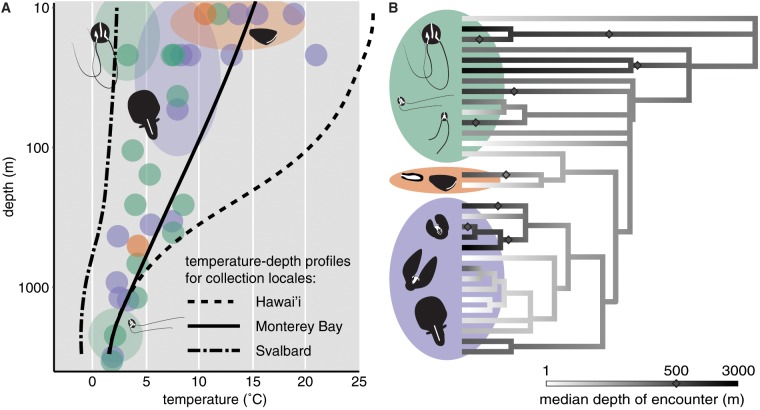
Ctenophore habitat diversity. (**A**) Median depth versus temperature for 31 species, overlaid with typical depth–temperature profiles for collection locales. The shading of each data point represents the order of Ctenophora to which that species belongs. Ovals show approximate distributions of pictured species: some, such as *B. infundibulum* and *B. forskalii* (the two animals lacking tentacles) have broader depth or temperature distributions than others. Overall range of the Ctenophora encompasses three main qualitative habitat types: shallow-warm, shallow-cold, and deep-cold. No species have yet been reported from deep hydrothermal vents. (**B**) Brownian motion ancestral reconstruction of habitat depth across 31 of the sequenced ctenophore species. Depth is mapped to a log scale as in **A**, in accordance with the adiabatic gas law. Transitions across 500 m are marked with diamonds to emphasize multiple convergent adaptive events (7 shallow→deep and 2 deep→shallow). All three orders of ctenophore (Cydippida; Lobata; and Beroida) are well-represented in shallow-warm, shallow-cold and deep-cold habitats. Cydippida is polyphyletic (Podar et al. 2001). This provisional phylogeny was inferred using the RAxML program on an alignment of 248 single-copy orthologous proteins. Note that Brownian Motion reconstruction is sensitive to taxon exclusion bias (Pennell et al. 2015): while it serves to propose convergent evolution scenarios for our analyses, it may not be adequate to confidently reconstruct the habitats of ctenophore ancestors.

Here, we demonstrate the power of such an analysis by applying an evolutionary model-based tool to metabolic enzyme alignments from a set of 34 different ctenophore transcriptomes. Ctenophores, having evolved repeatedly to colonize shallow, deep, warm, and cold habitats (Fig. 1), present an attractive opportunity to explore patterns of protein adaptation to hydrostatic pressure and to temperature. In particular, repeated evolution of environmental tolerance phenotypes allows us to dissect the convergent nature of these adaptations and assess the size of the adaptive solution space within which biochemical evolution works.

### Pressure and temperature: fundamental parameters of chemistry and biology alike

Each organism is a complex system of chemical reactions, with enzyme catalysts (Dong and Somero 2009) and substrates (Hochachka 1985) adaptively tuned to the environment that the organism inhabits. Pressure and temperature influence the rate and equilibrium of all chemical reactions, which is the principle underlying differences in environmental tolerance across species and motivating the study of pressure and temperature as evolutionary selectors. Effects of temperature on ectotherm metabolism are relatively well understood: enzymatic reaction rates track exponentially with environmental temperature, changing the respiratory rate in similar fashion until a pejus threshold is reached (pejus means “getting worse”). At an organism’s lower pejus temperature, enzymes’ unequal temperature dependencies critically disrupt the coupling of metabolic reactions; at the upper pejus temperature, proteins can unfold as well. Sustained exposure to pejus temperature causes respiration to drop precipitously and often kills the organism (Pörtner 2001). This relationship between metabolic flux, protein folding, and temperature subjects enzymes to an evolutionary trade-off resulting in “marginal stability,” a state in which an enzyme is rigid enough not to denature, but flexible enough to perform catalysis at its native temperature (Somero 2003). Cold-adapted enzymes tend to be highly efficient (high reaction velocity at low temperature) but unstable; vice versa for their warm-adapted orthologs (Fields et al. 2006; Lockwood and Somero 2012; Dong et al. 2018).

Metabolic effects of increased pressure have been generalized as mirroring those of low temperature (Menzies et al. 1974) and in some cases elevated temperature has been reported to rescue organisms from hyperbaric metabolic depression (Brown and Thatje 2011). In reality, organismal responses to pressure are highly taxon-specific and, like responses to temperature, may not be monotonic (Brown et al. 2017). Metabolic effects of both pressure and the temperature–pressure interaction vary in direction. At unnatural pressures, respiration may decrease (Bailey et al. 1994) or increase (Morita 1957; Bartlett 2002) and in some cases, behavior appears to be unaffected (Cottin et al. 2012). When organismal metabolism is separated into its constituent chemical reactions, the important but idiosyncratic properties of reaction coupling drop away, and pressure effects are explained simply by the volume change of the system (Somero 1990). Intuitively, reactions that increase in volume are inhibited by high pressure, whereas contractile reactions are enhanced. For a given enzymatic reaction, for example, pyruvate to lactate, the net volume change cannot be altered, but the transient volume changes associated with substrate binding and conversion (i.e., “binding volume” Δ*V*_E_ _+_ _S → E•S_ and “transition volume” Δ*V*^‡^) are often adjusted. These volumes tend to be minimized in deep-adapted enzyme orthologs (Low and Somero 1976). Strong metabolic responses to pressure at the organismal and single-reaction levels (Teal and Carey 1967), along with adaptive structural modifications in a variety of enzymes and cytoskeletal proteins (Siebenaller and Somero 1978; Somero and Siebenaller 1979; Swezey and Somero 1985; Morita 2008), demonstrate that hydrostatic pressure exerts considerable selective pressure. It is at least as pervasive a selector as temperature, since >98% of Earth’s habitable volume lies between 200 and 11,000 m ocean depth (Dawson 2012) and therefore under 20–1100 bar of pressure.

### Function to structure: sparsely charted waters

Since the unifying functional concepts of marginal stability and volume change were implicated in temperature- and pressure-adaptation of proteins, there has remained an open question as to how these characteristics map to protein-coding loci in the genome. Most progress has been made in the realm of thermal stability, where ubiquitous structural themes have been identified. These include substitution of uncharged amino acid residues with charged ones, which form hydrogen bonds to hold domains or subunits together (Dong and Somero 2009; Lockwood and Somero 2012) and substitution with bulky hydrophobic amino acids to facilitate stabilizing van der Waals interactions in the protein core (Haney et al. 1999). Substitution with proline can also introduce turns at critical sites in the protein backbone, limiting its flexibility (Bogin et al. 1998).

There are two leading structural hypotheses for pressure-adaptation of proteins. One involves hydrophilicity, positing that since water potential is higher under pressure, hydrophilic configurations are favored and pressure-tolerant proteins either (1) evolve to maximize surface hydration or (2) in the case of enzymes, evolve to minimize change in surface hydration between the free and substrate-bound conformations (Somero 1990). To a first approximation, this implies that high-pressure-adapted proteins should have more hydrophilic residues in contact with solvent than their low-pressure orthologs. Indirect support for this hypothesis comes from work with the small molecule trimethylamine N-oxide (TMAO), which protects proteins against pressure perturbation in deep-sea fishes (Yancey et al. 2004). TMAO alters the organization of water (Sharp et al. 2001) so that it more thoroughly hydrates macromolecules (Schroer et al. 2016). If this effect can stabilize proteins against pressure, it stands to reason that intrinsically hydrophilic surfaces might as well.

Another structural hypothesis concerns buried amino acid residues rather than solvent-exposed ones. It proposes simply that bulkier, less compressible amino acid side chains can fill voids in a protein’s interior, making it resistant to structural perturbation by pressure (Chalikian and Macgregor 2009). An analysis of globular protein compressibilities (Gromiha and Ponnuswamy 1993) supported this hypothesis by finding the strongest determinant of protein compressibility to be compressibilities of its constituent amino acids. The predictive model that arose from that study is an attractive step toward describing the structural basis of pressure tolerance, but requires improvement in two major areas. First, being composition-driven, the model neither account for the sequence context of amino acids nor effects of secondary structure, which are known to be significant (Gross and Jaenicke 1994). Second, its relevance to enzymes remains unclear because a link has yet to be established between adiabatic compressibility and biochemical properties like substrate binding affinity and maximal reaction velocity.

Thanks to next-gen sequencing, we are able to use a comparative transcriptomic approach to map organismal environmental tolerance phenotypes to protein-coding loci. As a comparative method, this requires sampling organisms across the broadest possible ranges of pressure and temperature. It is equally imperative that our comparative analysis take phylogeny into account and derive its power from multiple independent (i.e., convergent) adaptive events (Dunn et al. 2018). This approach should avoid “phylogenetic pseudoreplication” that affects phylogeny-agnostic methods (Garland 2001), where apparent statistical support is derived from multiple species even though they share homologs of a single adaptive innovation.

### Enter the ctenophore

Ctenophores, or “comb jellies,” are a clade of marine animals fulfilling both of the above requirements. They can be found living at −2°C to 30°C, from the surface to over 7000 m depth (Lindsay and Miyake 2007) (Fig. 1A), and their phylogeny reveals multiple independent colonizations of deep and shallow waters (Fig. 1B). In addition, as an early-diverging lineage with hundreds of millions of years of distinct evolutionary history (Dunn et al. 2015), they present an attractive opportunity to probe convergent molecular evolution on a grand scale by way of eventual comparison to Cnidaria. Having assembled transcriptomes for 34 ctenophore species, we have sufficient data both to infer a working ctenophore phylogeny and to analyze the amino acid sequences of pressure- and temperature-sensitive proteins. Experimental data verify that ctenophore enzymes exhibit specialized pressure responses that parallel non-ctenophore orthologs (cf. Low and Somero 1976). For example, pyruvate kinases (PKs) of the shallow-adapted ctenophores *Beroe forskalii* and *Lampea lactea* are kinetically inhibited nearly three-fold by high pressure, whereas the ortholog from an undescribed deep benthic ctenophore is pressure-enhanced up to ten-fold (Thuesen EV and Bachtel TS, unpublished data).

### Dissecting the layers of convergent adaptation

**Fig. 2:**
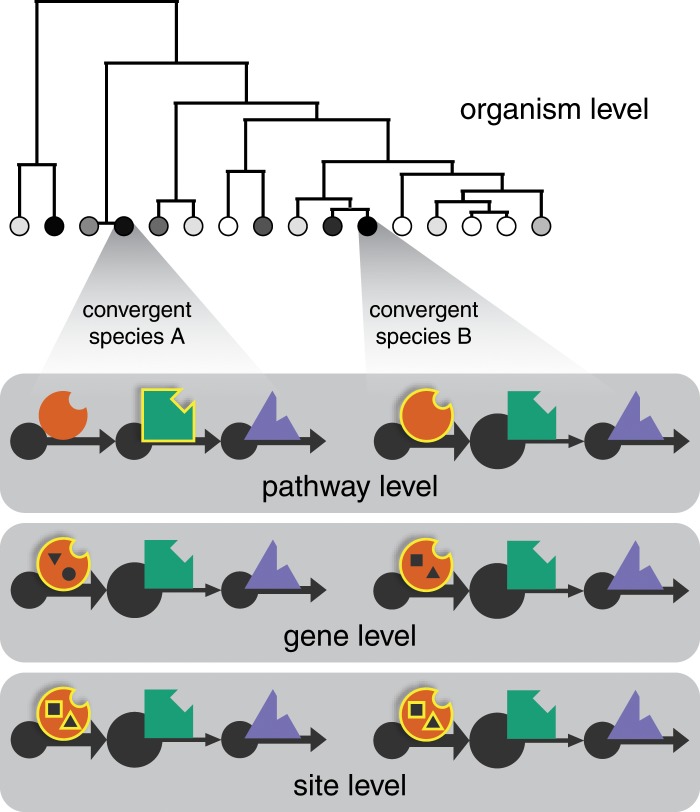
Levels of convergent adaptation. Convergent evolution of organismal traits can be caused by convergence at various sub-organismal scales. Three possible explanations for organism level convergence are illustrated here using a hypothetical metabolic pathway with three enzymes. Elements accounting for the convergent organismal trait in each scenario are highlighted and biochemical flux corresponds to arrow weight. Pathway level convergence can occur when a derived phenotype is produced by adaptations in the same pathway, but not necessarily the same gene. For example, in Species B, the first enzyme compensates for diminished binding affinity in the second enzyme by feeding it more substrate, whereas in Species A, the second enzyme has evolved a higher binding affinity to achieve comparable flux through the pathway. Gene level convergence implies that the same gene is responsible for the derived phenotype in both species, but the adapted sites within this gene, represented by shapes inside the cartoon of the first enzyme, are not necessarily the same in Species A and B. Site level convergence requires the same sites in the same gene to account for the derived phenotype, implying a small evolutionary solution space for the adaptive challenge in question.

From molecular phylogenies, it is apparent that several distantly related ctenophores are convergently adapted to live at similar depths (Fig. 1). Such homoplasy at the organismal level may be the most common meaning of “convergent adaptation,” but homoplasy likely extends further into the pathways and genes associated with environmental tolerance. Detecting convergence at the scale of amino acid sites allows us to resolve the level at which convergent adaptation is taking place. There are three different levels of convergence that could explain the same organism-level phenotypic pattern (Fig. 2). Consider a hypothetical environmental tolerance phenotype and a metabolic pathway featuring three enzymes. (1) Convergence can exist at the pathway level when a derived phenotype is produced by adaptations in the same pathway, but not necessarily in the same gene. For example, in Species B, the first enzyme compensates for decreased binding affinity in the second enzyme by feeding it more substrate, whereas this is unnecessary in Species A because the second enzyme has evolved a higher binding affinity. Total flux through the pathway is the same in both species. (2) Convergence at the gene level implies that the same gene is responsible for the derived phenotype in both species, but the adapted sites within this gene and its enzyme product are not necessarily the same in Species A and B. (3) Convergence at the site level requires the same sites in the same gene to account for the derived phenotype. Extensive site-level convergence implies a small evolutionary solution space for the adaptive challenge in question. Note that other authors (Stern 2013; Lee and Coop 2017) have referred to (1) above as convergent adaptation and (3) as parallel adaptation. Viewing convergence as a matter of degree rather than kind (Arendt and Reznick 2008) motivates the terms used here. All three of the above patterns contribute to other kinds of environmental adaptation (Riehle et al. 2001) and so may all play a role in the cases of pressure and temperature as well.

### Structure-functional hypotheses: needles in a transcriptomic haystack

Using a phylogenetically informed comparative method to resolve convergent adaptation at the amino acid site scale is conceptually straightforward, but impractical without post-genomic methods. Traditional comparative biochemistry would prescribe choosing pairs of orthologous proteins from differently-adapted congeners, then reciprocally mutating every residue that differs between the orthologs and assaying the mutants under relevant environmental conditions (Morita 2008). With 60–100 sites differing for a pair of typical 500-residue globular proteins, this would amount to a heroic mutagenesis effort (Kazlauskas and Bornscheuer 2009). Expanding the scope of convergence to consider multiple interacting proteins becomes intractable. Transcriptome analysis alleviates this problem by identifying a manageable number of high-likelihood hypotheses that can be tested by site-directed mutagenesis. Once a convergence-detection algorithm has been experimentally validated at the site scale, it can be applied to complete transcriptomes to identify additional genes associated with a phenotype and to assess the multi-gene architecture of convergent phenotypes as discussed earlier.

## Methods

### Specimen and data collection

Ctenophores were collected using blue water SCUBA techniques (0–20 m depth) and Remotely Operated Vehicles Ventana and Doc Ricketts (200–4000 m depth). Median depth and temperature of occurrence was determined for each species using the MBARI Video Annotation and Reference System database (Schlining and Jacobsen Stout 2006). Median was used because many depth distributions are strongly skewed. For species not collected on MBARI expeditions, temperature was estimated using temperature/depth data from the collection locale. The log_10_(depth) was used in our analyses, consistent with similar previous investigations (Porter et al. 2016) and with the adiabatic gas law.

Primary metabolism was chosen as the biological system on which to test our hypotheses. Metabolic pathways like glycolysis and the Krebs cycle are essential to survival and involve a set of known interactions between well-characterized enzymes. Many of these enzymes exhibit pressure effects in other marine taxa (Siebenaller and Somero 1978; Somero and Siebenaller 1979; Dahlhoff and Somero 1991; Gerringer et al. 2017). Amino acid sequences for the enzymes D-lactate dehydrogenase (LDH, EC 1.1.1.28), cytosolic and mitochondrial malate dehydrogenase (cMDH and mMDH, EC 1.1.1.37), and PK (EC 2.7.1.40) were aggregated using OrthoFinder (Emms and Kelly 2015) from 34 transcriptomes assembled using Trinity (Haas et al. 2013) and translated with TransDecoder (Haas et al. 2013). Transcriptomes were derived from samples containing comb row tissue, since this tissue exhibits high activity for all the above enzymes (Thuesen EV, unpublished data). The guide phylogeny for comparative analysis, a subset of which is shown in Fig. 1, was inferred using the RAxML program (Stamatakis 2014) on an alignment of 248 single-copy orthogroups.

### Site-level convergence detection

Convergently adaptive sites were predicted using the Profile Change with One Change (PCOC) algorithm (Rey et al. 2018). In brief, this method works by applying a 60-category siteheterogeneous (CAT) model of protein evolution in a maximum likelihood framework (Quang et al. 2008) along a species tree and determining, on a site-wise basis, whether branches designated as having a “convergent” phenotype conform to a different amino acid profile than branches with the “ancestral” phenotype. Also incorporated in PCOC is the frequency of amino acid changes on transition branches between the ancestral and convergent phenotypes. To accommodate our analysis of continuous environmental tolerance phenotypes, extensions were made to the existing PCOC workflow and implemented as a Python wrapper script available here: github.com/octopode/continuous-converge.

### Convergent scenario formulation

**Fig. 3:**
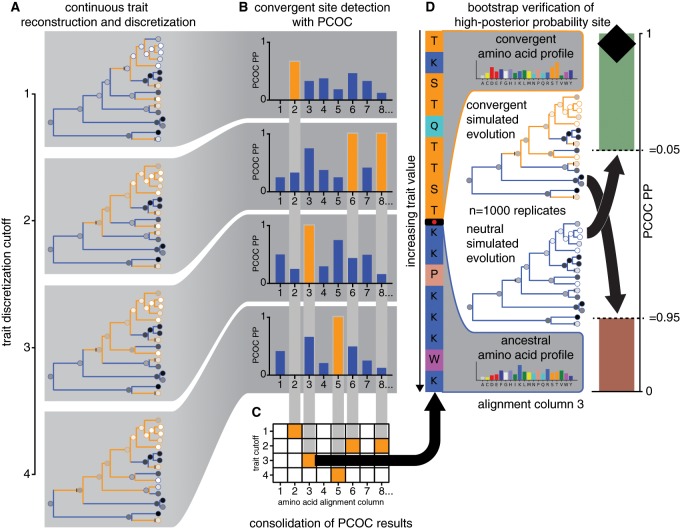
Schematic of PCOC algorithm implementation for a continuous trait. The analysis begins with convergent scenario formulation (**A**). Dark blue and light orange represent “ancestral” and “convergent” states here; they also correspond to the predominant amino acids in the ancestral and convergent parts of the alignment column shown. Considering all convergent scenarios, the script proceeds to identify the highest-PP trait cutoff at each site (**B, C**) and finally runs a bootstrap test on a high-PP site (Site 3 shown) to yield PP confidence thresholds as reported in a column of the Manhattan plot (**D**). Where high PP scores occur frequently by chance, that is, through neutral simulated evolution, the threshold for calling the site adaptive increases. PP (represented by the black diamond) within the upper/green area indicates a convergently adaptive site with bootstrap support of ≥95%, PP within the lower/red area indicates a not convergently adaptive site with bootstrap support of ≥95%, and PP in the middle/white area or in the overlap of the upper and lower shaded areas indicates unresolved site status.

The PCOC method as published requires the investigator to reconstruct the trait of interest along the species tree using binary states. This is straightforward in the case of a naturally dichotomous character like C4 metabolism in plants (Rey et al. 2018), but non-trivial when dealing with continuous characters like temperature and depth of occurrence. We used the following routine to propose convergent scenarios consistent with trait values and phylogeny:

First, a simple ancestral trait reconstruction was performed along the species tree as in Fig. 1. Trait values (temperature or log_10_(depth)) were recorded for each internal and terminal node, then binned using a set width of 1.75°C or 0.2 log_10_(m). Finally, a trait cutoff was placed between each pair of adjacent bins and each cutoff was used to place a set of transition events on the tree (as in Fig. 3A), comprising a “convergent scenario” in which the continuous trait of interest was rendered binary. Because PCOC only registers transitions in one direction, this binary trait was inverted for all scenarios in which the root reconstructed as “convergent.” In biological terms, this amounted to calling the “shallow” or “cold” state (trait values less than the cutoff) convergent and the “deep” or “warm” state (trait values greater than the cutoff) ancestral. We entertained both possibilities because reconstructing the actual habitat of the ctenophores' last common ancestor is outside the scope of this analysis. The resultant set of scenarios was filtered to ensure that each was unique and that a minimum of five independent transition events were present. The latter mitigated false positives due to phylogenetic pseudoreplication. For each enzyme alignment, the PCOC algorithm was repeated under 18 depth convergence scenarios and 6 temperature convergence scenarios, each with a different trait cutoff (as in Fig. 3B). The maximum PCOC posterior probability (PP) across all scenarios was recorded for each site, provided that it fell above a threshold of 0.5 (as in Fig. 3B and C). This rule was used to reduce computational load in the following step.

### Evolutionary simulation-based bootstrapping

The PP value returned by PCOC gives a likelihood-based relative measure of whether an amino acid site evolved convergently in association with a phenotype. It does not provide a controlled level of statistical confidence. As this extra measure of confidence is desirable for designing mutagenesis experiments, we used the simulation feature included in the PCOC toolkit to implement an automatic bootstrap test (Fig. 3D). For each site, the maximum-likelihood CAT amino acid profile was recovered for both the ancestral and convergent branches. The CAT model was then used to repeatedly simulate evolution along the species tree. To obtain false positive rate α (1 - specificity), non-convergent evolution was simulated using only the ancestral profile with noise added. To obtain false negative rate β (1 - sensitivity), convergent evolution was simulated using both profiles, with transitions placed according to the maximum-PP convergent scenario. One thousand of each non-convergent and convergent simulations were run per site.

This bootstrap test improves upon existing PCOC simulation facilities by providing positive and negative calling confidence thresholds based on a more site-specific model. Where the previous tool allowed simulation of non-convergent and convergent evolution along the actual species tree using random pairs of amino acid profiles, our extension specifies the profiles of best fit to the alignment column in question. If more conservative bootstrapping is desired, it still permits incorporation of branch-length uncertainty and other sources of noise into the model.

### Protein structural analysis

Though PCOC has yet to be empirically validated with respect to temperature- and pressure-adaptation, we performed some simple hypothesis tests downstream of adaptive site prediction to illustrate the types of structure–functional relationships comparative transcriptomic analysis can be used to explore. B-factor profile, a measure of predicted local conformational mobility, was transformed using Ordered Quantile Normalization and modeled as a function of predicted depth- and temperature-adaptiveness using two-way ANOVA (Type III sum of squares). To ascertain biases in secondary structure composition and local environment of predicted adaptive sites, Fisher’s exact test was used with “expected” site counts corresponding to the distribution of predicted non-adaptive sites. Unresolved sites were omitted from the analysis because they are subject to sampling bias: inconclusivity is an artifact caused by insufficient taxon sampling. All statistical tests were performed in R. To model 3D structures from which B-factor profile, secondary structure, and local environment data could be extracted, the the I-TASSER server suite (Roy et al. 2010) was applied to enzyme sequences from *Bolinopsis infundibulum*. The crystal structure used as a threading template for LDH was manually set to PDB: 1F0X to ensure that the template’s EC number agreed with that of the query protein.

## Results

### Frequency of adaptive sites, overlap of pressure- and temperature-adaptation

**Fig. 4:**
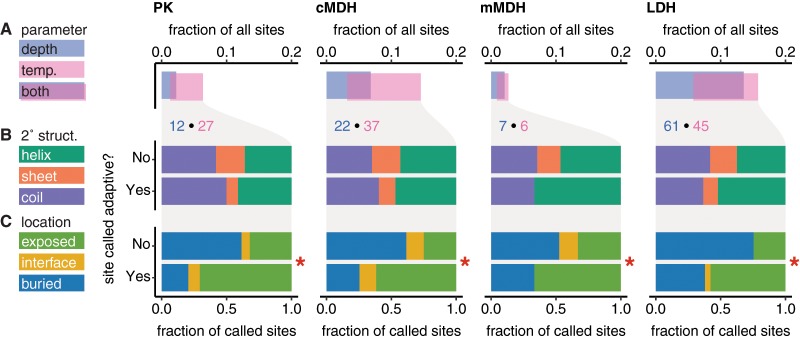
Comparison of adaptive and non-adaptive sites across PK, cMDH, mMDH, and LDH. Total numbers of depth- and temperature-adaptive sites in each enzyme, as well as their degree of overlap, are shown in **A**. Distribution of sites are considered among helix, sheet, and coil secondary structures (2˚ struct., **B**) and among solvent-exposed, subunit interface and buried locations (**C**). Sites in each of these enzymes predicted by PCOC to be adaptive to depth or temperature, that is, “called adaptive” are compared to those predicted to be adaptive to neither parameter. Comparisons found to be significant to a level of α < 0.05 are marked with an asterisk. Depth- and temperature-adaptive sites exhibited similar distributions in each enzyme; these distributions are presented separately in Supplementary Fig. S1.

Convergent adaptation associated with habitat depth was predicted at 2.0–13.5% of sites in each enzyme. Convergent adaptation associated with temperature was predicted at 1.8–11.5% of sites and 0.9–7.8% of sites were associated with both environmental parameters. Across all four enzymes, 46 sites were associated with depth-adaptation, 59 with temperature-adaptation, and 56 with both. For brevity, sites predicted by PCOC to be adaptive to depth or temperature are referred to here as adaptive sites. There was considerable variation between enzymes, with LDH exhibiting the highest proportion of adaptive sites, and mMDH the lowest (Fig. 4A).

### Local environment of adaptive sites

**Fig. 5:**
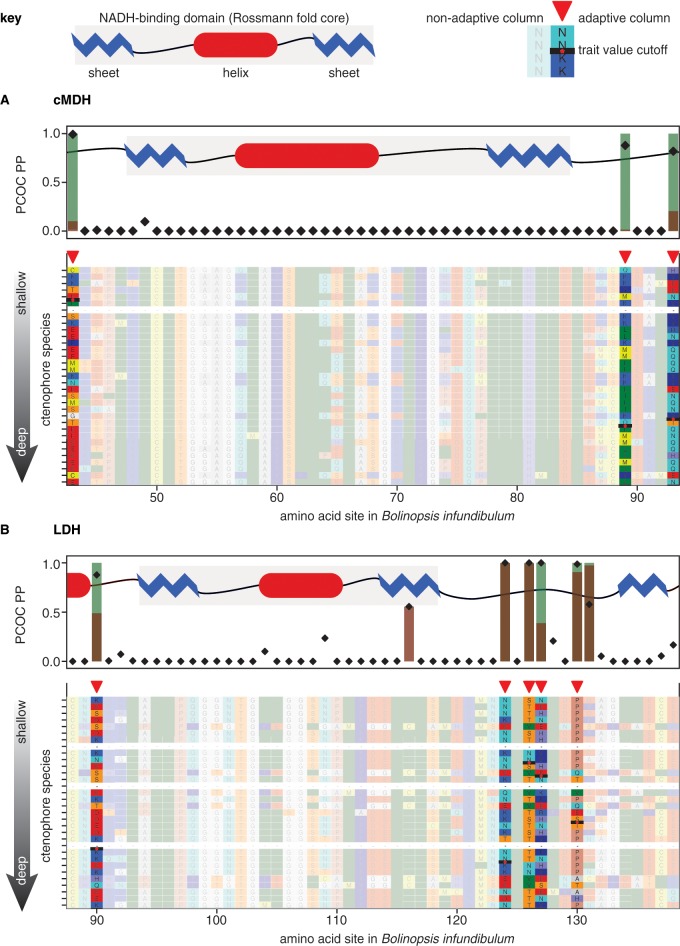
Alignment excerpts annotated with convergently adaptive sites. Alignment blocks contain the N-terminal NADH-binding domains of cMDH (**A**) and LDH (**B**) enzymes. While the core sheet–helix–sheet Rossmann fold regions, superimposed as cartoons, are well-conserved and feature no adaptive sites, depth-adaptive sites do appear in coils immediately flanking these motifs. These sites are highlighted and indicated with a red pointer. PCOC PP and bootstrap confidence thresholds are plotted above the alignment as in Fig. 3D. Complete alignments of all four enzymes, including species names, are included as Supplementary Fig. S2–5.

The distribution of predicted adaptive sites within predicted enzyme structures was consistent across enzymes. Adaptive sites were overrepresented in solvent-exposed regions (Fig. 4C), but no bias was found in their distribution among helix, sheet, and coil secondary structures (Fig. 4B). In LDH, depth-adaptive sites occurred in significantly more flexible parts of the protein as reflected in B-factor profile (Supplementary Fig. S1B), whereas temperature did not correlate significantly to B-factor profile in any of the four enzymes. Residue counts and more detailed statistics can be found in Supplementary Table S1. In cMDH and LDH, several adaptive sites were predicted flanking the NADH binding domain, a Rossmann fold motif near the N-terminus of the dehydrogenases (Fig. 5). Complete adaptive site maps of all four enzymes with respect to both environmental parameters analyzed can be found in Supplementary Materials (Supplementary Figs. S2–5).

## Discussion

### Consistency with historical high-pressure enzymology results

The four enzymes analyzed here were chosen because they are essential to primary metabolism and because their functional responses to pressure have been characterized in other taxa. PK of shallow ctenophores and other animals exhibits decreased maximal velocity under pressure, but the mechanism for this is obscure beyond that the enzyme has positive activation volume, undergoing a transient expansion to convert its substrate (Low and Somero 1976). At around 1000 bar, subunits of rabbit PK are known to dissociate (de Felice et al. 1999), but absence of depth-adaptive sites in the subunit interface suggests that this inhibition mechanism may not affect ctenophores under biologically relevant pressures. Lactate and malate dehydrogenase (LDH and MDH) are both NAD^+^-dependent dehydrogenases. Members of this enzyme family are highly pressure-sensitive in shallow-living fishes (Somero and Siebenaller 1979), crustaceans, molluscs, and annelids, due in part to pressure-inhibited binding of their cofactor NADH (Somero and Siebenaller 1979; Dahlhoff and Somero 1991). The Rossmann fold domain responsible for binding this cofactor constitutes a sheet–helix–sheet motif within about 100 residues of each enzyme’s N-terminus. Depth-adaptive sites were predicted adjacent to the Rossmann fold in in all three dehydrogenases analyzed. None of the depth-adaptive sites were in direct contact with NADH; contact amino acids are highly conserved, but sites flanking the Rossmann fold could affect its compressibility and thus its ability to bind NADH under pressure.

### Potential implications for environmental adaptation of proteins

Large variation in proportion of adaptive sites among just four enzymes suggests that site-level convergence occurs in depth- and temperature-adaptation, but that convergence may occur at higher levels as well (Fig. 2). There are two possible explanations for the large overlap of sites predicted to be adaptive to pressure and temperature. First and most conservative is that depth and temperature of encounter covary too strongly in our dataset. In this case, sampling greater temperature diversity among shallow taxa will better resolve sites adapted to each environmental parameter and may decrease their overlap. Second, it is possible that these sites are actually adapted to both pressure and temperature, or adapted to one and exapted to the other. If expanded taxon sampling fails to support the first explanation, then the second must be tested experimentally (see further work).

Three trends emerged from analysis of adaptive sites’ local environment in the protein. First, adaptive sites rarely exhibited a bias in conformational flexibility, that is, B-factor profile. Second, the secondary structure composition of adaptive sites was not significantly different from that of non-adaptive sites. Third, adaptive sites were more likely than non-adaptive sites to be in contact with solvent and in LDH, all subunit interface sites were predicted to be adaptive, despite the presence of only three interface residues per subunit. This is reflected by lack of a yellow bar in the “No” row under LDH in Fig. 4C.

The analysis of B-factor profile was intended to address convergent evolution of marginal stability. Observed lack of differences in conformational flexibility between adaptive and non-adaptive sites has at least two plausible explanations. It could be that the “buffering” behavior of the marginal stability tradeoff causes convergently adaptive sites to occur at both critically flexible and critically rigid sites in roughly equal proportion. This predicts symmetric bimodal distribution of adaptive sites along the B-factor axis, which was not identified here but could emerge from analysis of many more enzymes. It is also possible that convergent evolution of marginal stability simply does not occur at the site level in these enzymes, suggesting a larger adaptive solution space for this general structural requirement than for other functions like ligand binding.

The lack of secondary-structural bias among adaptive sites fails to support observations that increased pressure favors formation of the α-helix over the β-sheet (Gross and Jaenicke 1994; Vajpai et al. 2013; Cieslik-Boczula 2017). If this phenomenon is evolutionarily relevant, one might expect to find disproportionately many depth-adaptive sites in vulnerable β-sheet regions. We did not observe a strong signal in the opposite direction, and it remains possible that pressure adaptation has a β-sheet bias in larger protein sets. The finding that adaptive sites are disproportionately common on the exterior of protein subunits was unsurprising, since buried residues tend to be more conserved. Still, it underscores that hydration and ligand interactions may be some of the more pressure-sensitive aspects of enzymes and raises the possibility that in these proteins, compression of internal voids either plays a secondary role, or else is a structural problem with many divergent adaptive solutions. All of the patterns identified here can be more rigorously evaluated and generalized by applying the same analysis to a much larger orthoset.

### Applications: from evolutionary biology to protein engineering

Environmental tolerances of proteins and pathways are of obvious interest to biologists seeking to understand organisms’ distributions in space and time. Abiotic factors like temperature and pressure often dictate a species’ fundamental niche. Knowledge of the number and type of mutations required to access a particular niche could lead to a better understanding of evolutionary lineages’ ecological history. It could also help to model how quickly various lineages might be able to adapt with global change.

Beyond basic science, temperature and pressure tolerance of proteins is a focus of biotechnology. Surveying the thermal diversity of life has yielded such popular biotech tools as thermostable *Taq* polymerase and heat-labile Shrimp Alkaline Phosphatase. Many chemical synthesis processes are conducted under pressure (Kunugi 1992), so exploration of bathyal diversity could yield analogous advances. At present, however, bioengineers are largely limited to the proteins that natural and artificial selection provide. They can optimize these proteins for various applications using rational design, but extensive structural knowledge is needed to make mutations that maintain function, let alone adjust it toward a specification. Molecular convergence-detection methods can narrow the vast parameter space associated with site-directed mutagenesis. With just an alignment and phylogeny from an appropriate set of taxa, a protein engineer might generate a tractable number of candidate mutations to achieve a desired behavior at particular temperature, pressure, pH, reactive oxygen species level, etc.

### Further work

It is essential that comparative transcriptomic techniques continue to be developed, tested, and refined. While the method reported here expands upon an innovative and promising technique introduced just last year, it can already be improved in several ways: first, convergent scenario formulation should be refined using a more sophisticated trait evolution model than Brownian motion. While it provides a useful approximation of ancestral traits, numerous problems with this model have been identified (Pennell et al. 2015). As implemented, it does not account for uncertainty in trait values and may facilitate placement of spurious convergent trait transitions. Though PCOC was found to be fairly robust to such errors (Rey et al. 2018), they increase the risk of false positives in principle. Second, when working with a transcriptome-scale dataset, it is appropriate to train the evolutionary model being used (in this case, the CAT model) on the dataset itself, rather than some external large alignment (Quang et al. 2008). Third, it would be useful to apply an evolutionary model with deletion and insertion parameters. PCOC uses tree pruning to handle gapped sites, but this strategy is not able to predict convergently adaptive insertions or deletions, features that are biologically interesting and useful to protein engineers.

At this point, comparative transcriptomic analyses are best used as powerful hypothesis generators. Algorithms like PCOC can identify patterns that have a very low likelihood of arising by chance, yet there are myriad covarying selective factors that could lure the overzealous investigator into a correlation-causation pitfall. Only controlled experiments can mitigate this risk and ultimately validate any predictive method. To this end, we will mutate predicted adaptive sites in the enzymes analyzed here and assay the mutant gene products across gradients of pressure and temperature. If these mutations cause the expected changes in pressure and temperature tolerance, then groundwork will be laid to apply comparative transcriptomics to countless other dimensions of biochemical diversity.

## Supplementary Material

icz063_Supplementary_Data
