# Supplementary material for: Combing Transcriptomes for Secrets of Deep-Sea Survival: Environmental Diversity Drives Patterns of Protein Evolution
Source: Integr Comp Biol. 2019 May 29;59(4):786–98. doi: 10.1093/icb/icz063 (PMC6797910; doi:10.1093/icb/icz063)
Supplement: icz063_Supplementary_Data [file icz063_supplementary_data.zip › icb-2019-0105-File007.docx]

**Combing transcriptomes for secrets of deep-sea survival:**

**Environmental diversity drives patterns of protein evolution**

J.R. Winnikoff, W.R. Francis, E.V. Thuesen, and S.H.D. Haddock

***Captions for Supplementary Materials***

Table S1: Raw counts and statistical summaries of adaptive sites

Table containing raw counts of adaptive and non-adaptive sites for the four enzymes examined, plus test parameters and statistics for assessing various biases in the distribution of adaptive sites throughout each enzyme’s structure. p-values less than 0.05 are shown in bold.

Figure S1: Detailed comparison of adaptive and non-adaptive sites across four enzymes

A comparison of the local environments of adaptive and non-adaptive sites within the enzymes pyruvate kinase (PK), cytosolic malate dehydrogenase (cMDH), mitochondrial malate dehydrogenase (mMDH) and lactate dehydrogenase (LDH). Sites called adaptive to depth, temperature or both parameters are compared to those called adaptive to neither parameter. Comparisons found to be significant to a level of α < 0.05 are labeled with red text. Total numbers of adaptive sites in each enzyme are shown in panel A; note that “both” is presented here as its own category. Distribution of sites is considered across a range of backbone mobility (B-factor profile values, panel B), among helix, sheet and coil secondary structures (2˚ struct., panel C) and among solvent-exposed, subunit interface and buried locations (panel D). The boxes in panel B represent first and second quartiles and the whiskers span maximum and minimum non-outlier values. Outliers are defined as falling more than 1.5 IQRs from the hinge.

Figures S2-5: Full adaptive site prediction results for the enzymes PK, cMDH, mMDH and LDH

Depth-adaptive sites are shown in panel A, temperature-adaptive sites in panel B. The Manhattan plots (panels A1 and B1) indicate PCOC posterior probability (PP) score for each amino acid site and whether it passes bootstrap verification as an adaptive or non-adaptive site. PP (represented by the black diamond) within the green area indicates a convergently adaptive site with bootstrap support of ≥95%, PP within the red bar indicates a non-convergently adaptive site with bootstrap support of ≥95% and PP in the white area or in the overlap of red and green indicates unresolved site status. In the amino acid alignments (panels A2 and B2), adaptive columns are highlighted and indicated with a red pointer. Taxa are sorted shallow→deep and warm→cold in panels A and B. The black partition with red dot in the each adaptive column indicates the trait discretization cutoff used to propose the convergent evolutionary scenario. The procedure used to predict adaptive sites is outlined in Figure 3.
