# Supplementary material for: Combing Transcriptomes for Secrets of Deep-Sea Survival: Environmental Diversity Drives Patterns of Protein Evolution
Source: Integr Comp Biol. 2019 May 29;59(4):786–98. doi: 10.1093/icb/icz063 (PMC6797910; doi:10.1093/icb/icz063)
Supplement: icz063_Supplementary_Data [file icz063_supplementary_data.zip › icb-2019-0105-File008.pdf]

secondary structure

	PK				cMDH				mMDH				LDH			
	**neither**	**depth**	**temp.**	**both**	**neither**	**depth**	**temp.**	**both**	**neither**	**depth**	**temp.**	**both**	**neither**	**depth**	**temp.**	**both**
**Coil**	210	3	11	3	97	5	9	5	117	1	0	2	159	11	7	8
**Helix**	180	3	9	2	119	4	12	6	153	2	2	2	142	11	3	23
**Sheet**	111	1	2	0	60	1	4	1	59	0	0	0	78	4	0	4
**Total**	501	7	22	5	276	10	25	12	329	3	2	4	379	26	10	35

PK: Fisher's $p = 0.75$; cMDH: Fisher's $p = 0.89$; mMDH: Fisher's $p = 0.89$; LDH: **Fisher's $p = 0.02$***

residue location

	PK				cMDH				mMDH				LDH			
**Buried**	308	2	5	0	170	2	7	3	173	1	1	1	286	13	3	11
**Exposed**	161	5	15	4	69	7	14	8	109	2	1	3	93	12	6	23
**Interface**	32	0	2	1	37	1	4	1	47	0	0	0	0	1	1	1
**Total**	501	7	22	5	276	10	25	12	329	3	2	4	379	26	10	35

PK: **Fisher's $p = 7.6E-5$***; cMDH: **Fisher's $p = 6.1E-5$***; mMDH: Fisher's $p = 0.59$; LDH: **Fisher's $p = 5.2E-10$***

B-factor profile

	PK				cMDH				mMDH				LDH				
	**SS**	**df**	**F**	***p***	**SS**	**df**	**F**	***p***	**SS**	**df**	**F**	***p***	**SS**	**df**	**F**	***p***	
**Depth**	1.46	1	1.49	0.22	0.07	1	0.07	0.79	0.25	1	0.26	0.61	7.22	1	7.61	**0.01**	*
**Temp.**	0.08	1	0.08	0.78	2.26	1	2.36	0.13	0.14	1	0.14	0.70	0.04	1	0.05	0.83	
**Depth x Temp.**	0.66	1	0.67	0.41	0.03	1	0.03	0.87	0.05	1	0.05	0.82	3.22	1	3.40	0.07	.
**Residuals**	521.73	532.00			307.12	320.00			328.43	335.00			423.74	447.00			
