# Supplementary material for: Combing Transcriptomes for Secrets of Deep-Sea Survival: Environmental Diversity Drives Patterns of Protein Evolution
Source: Integr Comp Biol. 2019 May 29;59(4):786–98. doi: 10.1093/icb/icz063 (PMC6797910; doi:10.1093/icb/icz063)
Supplement: icz063_Supplementary_Data [file icz063_supplementary_data.zip › icb-2019-0105-File009.pdf]

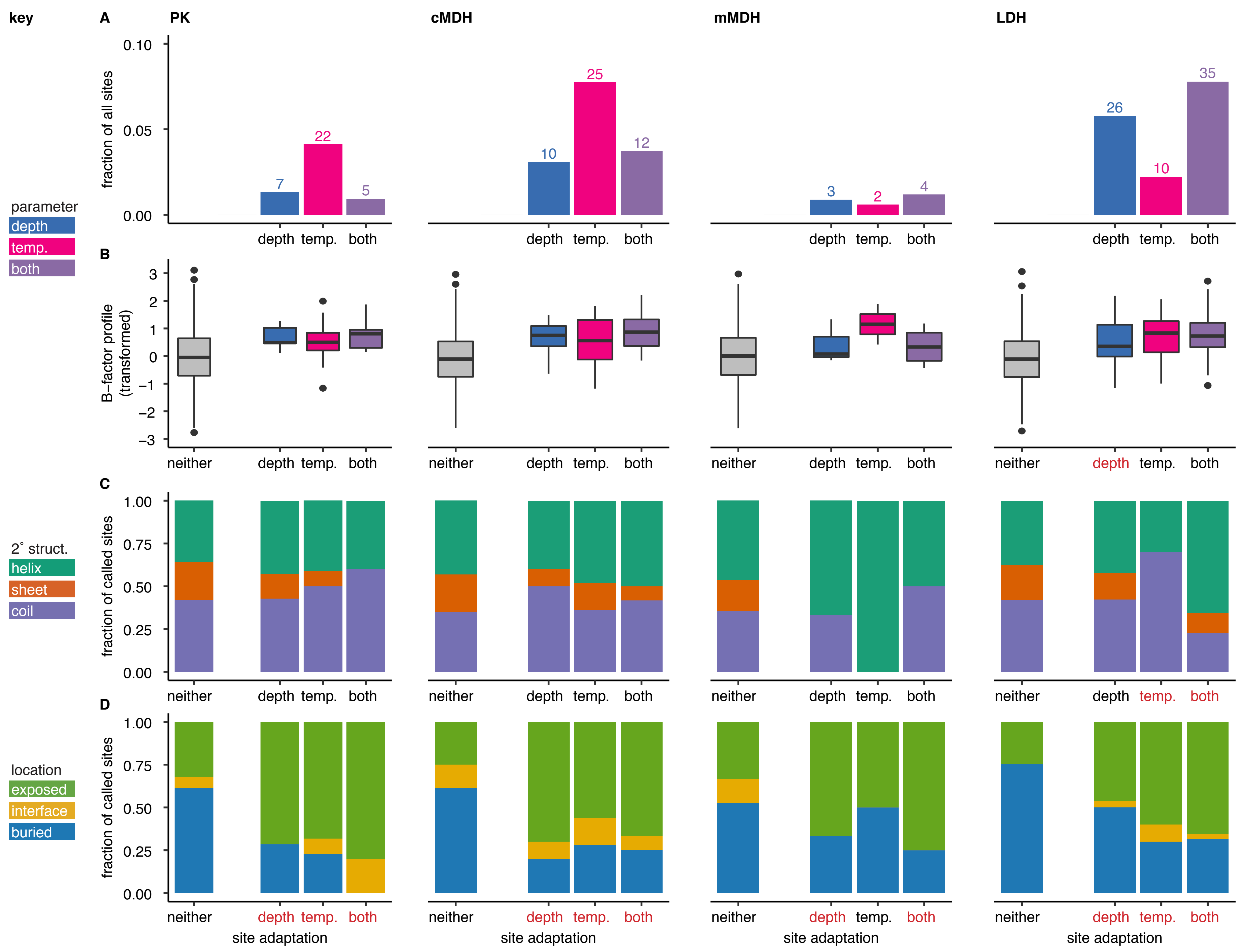

key
A
PK
cMDH
mMDH
LDH
fraction of all sites
0.10
0.05
0.00
7
22
5
10
25
12
3
2
4
26
10
35
parameter
depth
temp.
both
B
B−factor profile (transformed)
3
2
1
0
−1
−2
−3
neither
depth
temp.
both
C
fraction of called sites
1.00
0.75
0.50
0.25
0.00
2° struct.
helix
sheet
coil
D
location
exposed
interface
buried
site adaptation
