# Supplementary figures and images for: Combing Transcriptomes for Secrets of Deep-Sea Survival: Environmental Diversity Drives Patterns of Protein Evolution
Source: Integr Comp Biol. 2019 May 29;59(4):786–98. doi: 10.1093/icb/icz063 (PMC6797910; doi:10.1093/icb/icz063)

# pyruvate kinase (PK) - 34 taxa

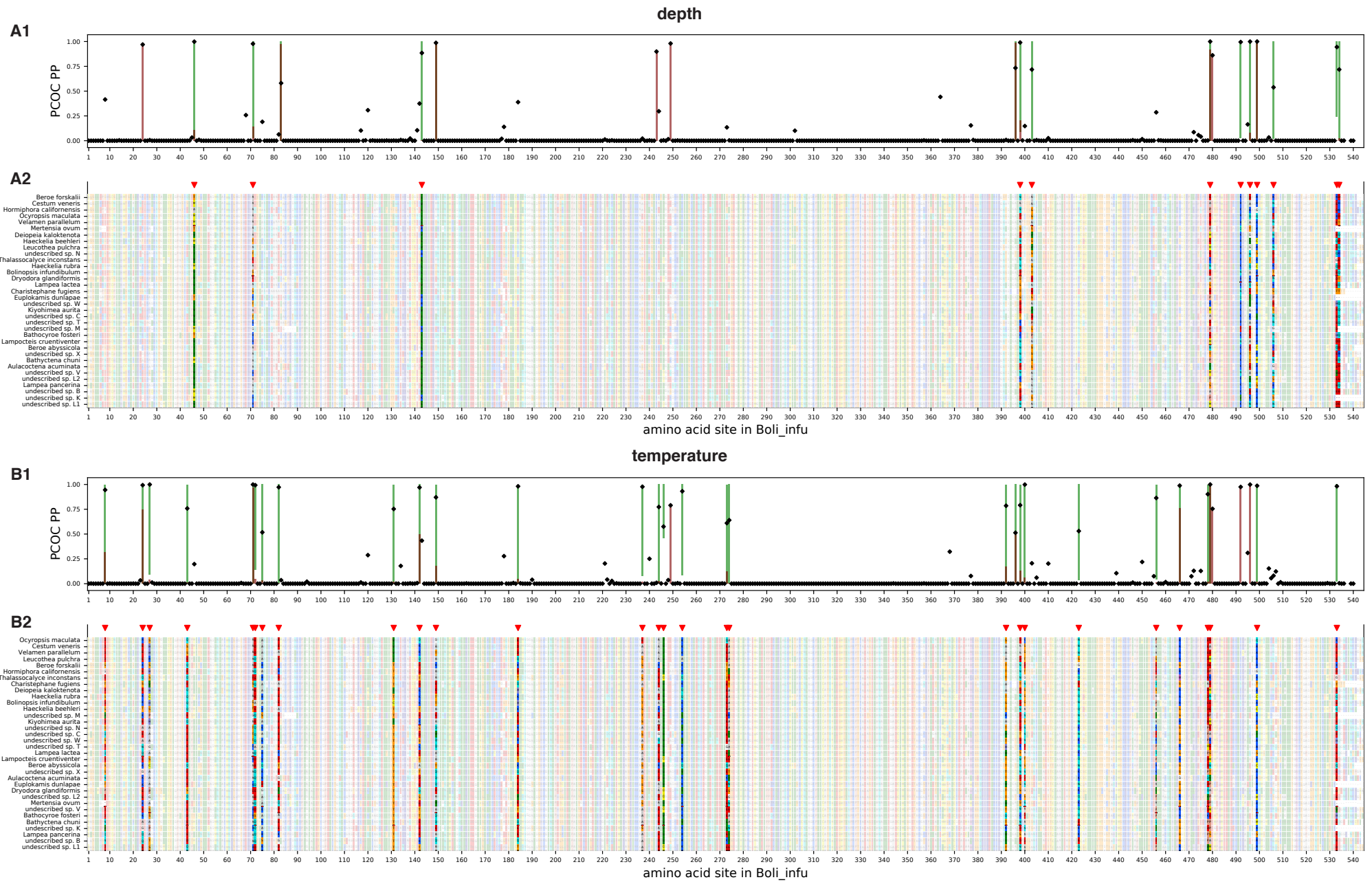

Supplement: icz063_Supplementary_Data [file icz063_supplementary_data.zip › icb-2019-0105-File010.pdf]

# cytosolic malate dehydrogenase (cMDH) - 33 taxa

depth

A1

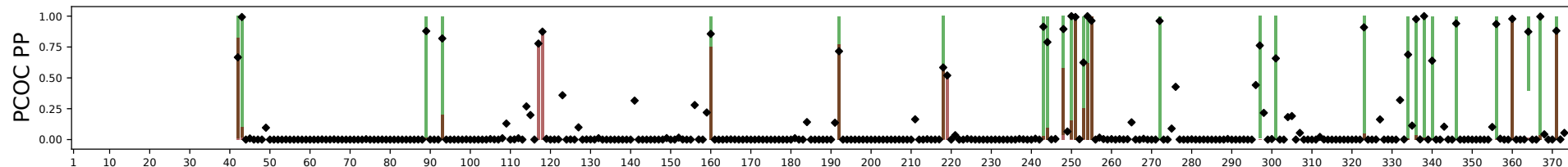

A2

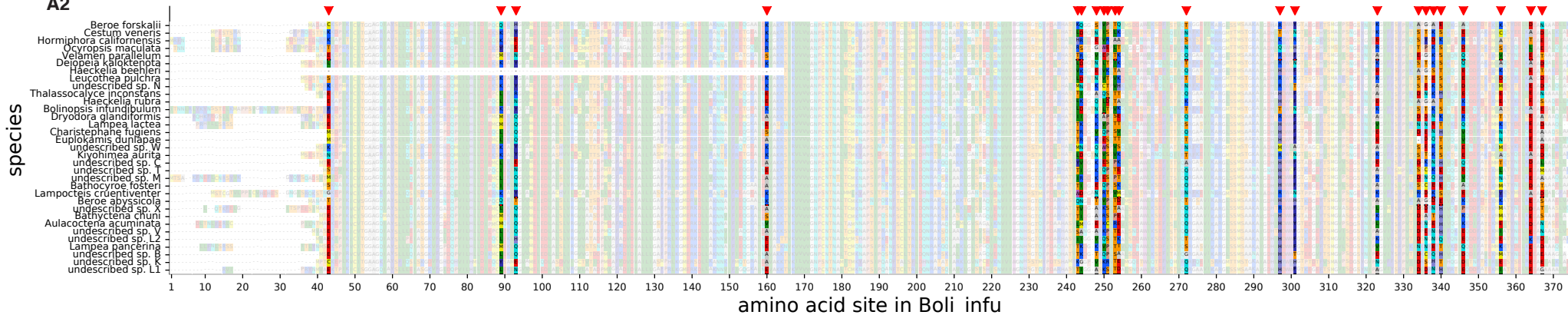

temperature

B1

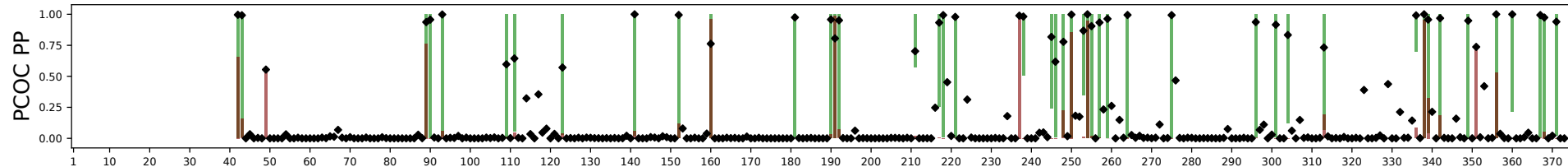

B2

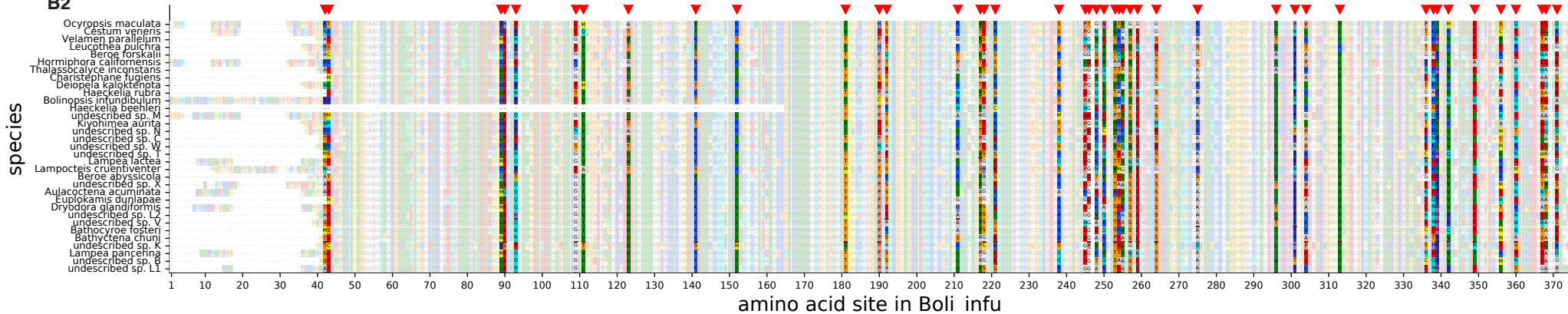

Supplement: icz063_Supplementary_Data [file icz063_supplementary_data.zip › icb-2019-0105-File011.pdf]

## mitochondrial malate dehydrogenase (mMDH) - 33 taxa

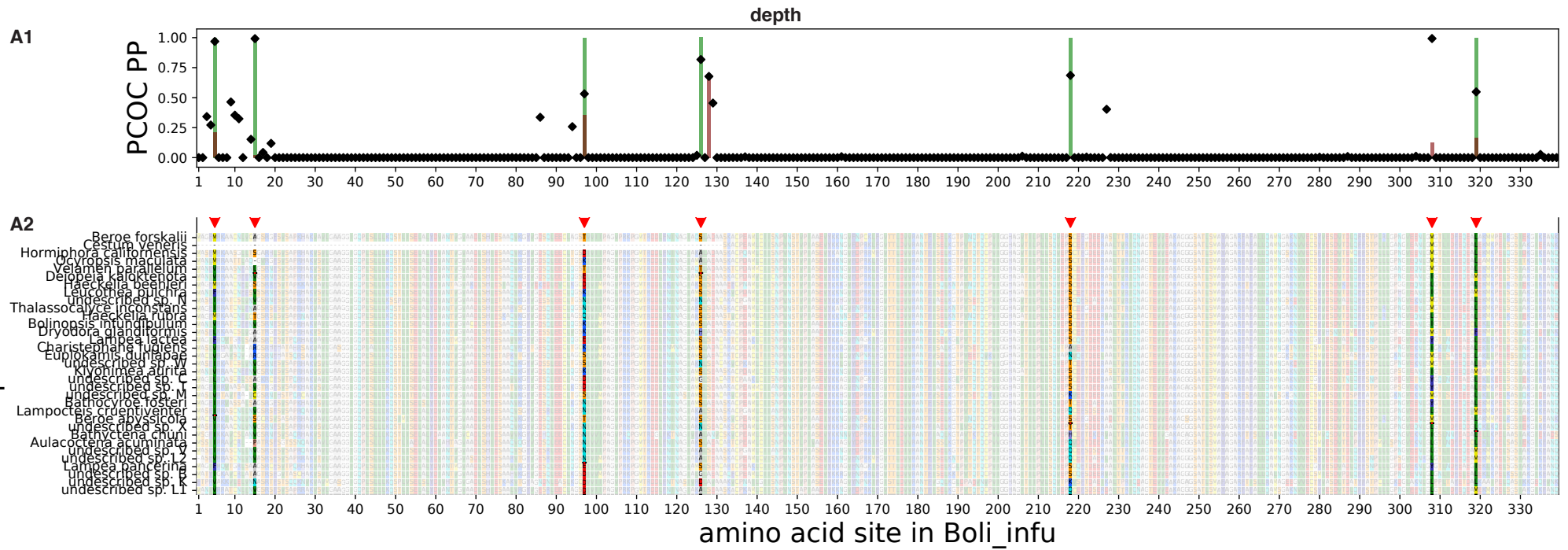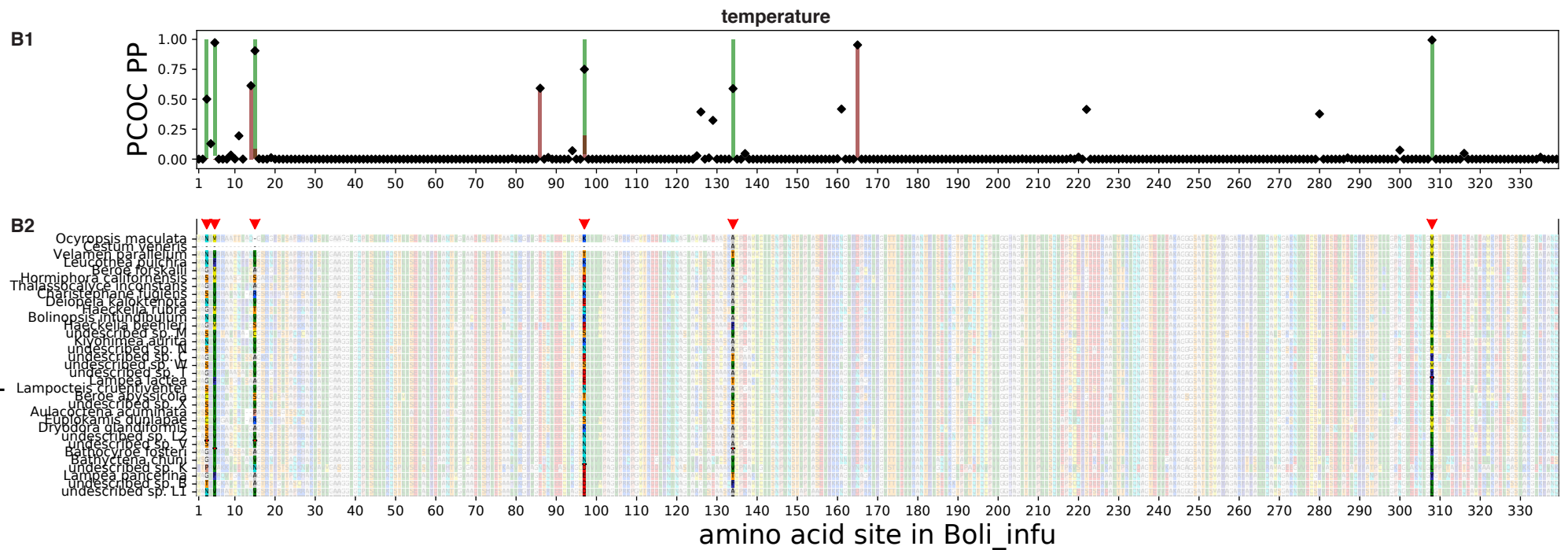

Supplement: icz063_Supplementary_Data [file icz063_supplementary_data.zip › icb-2019-0105-File012.pdf]

# D-lactate dehydrogenase (LDH) - 32 taxa

depth

A1

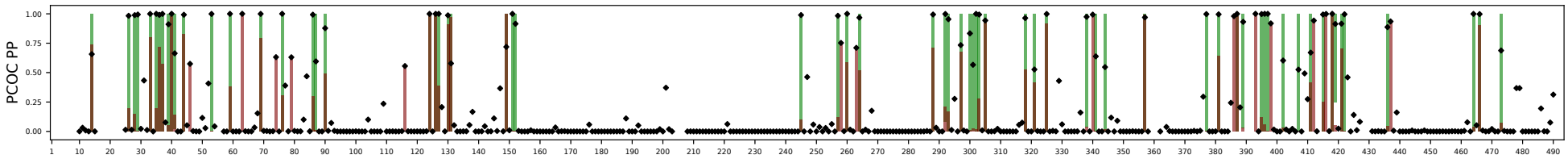

A2

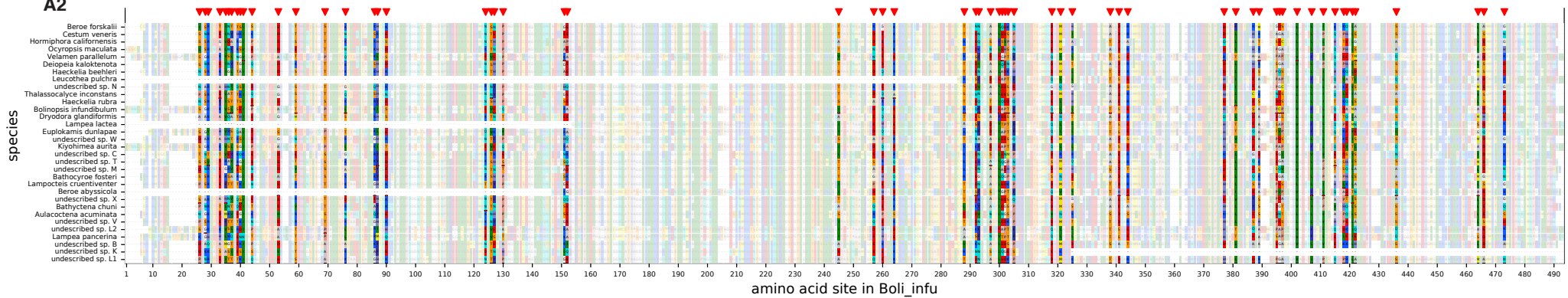

temperature

B1

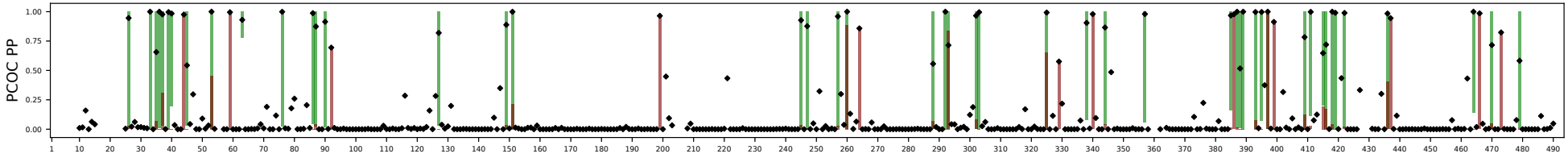

B2

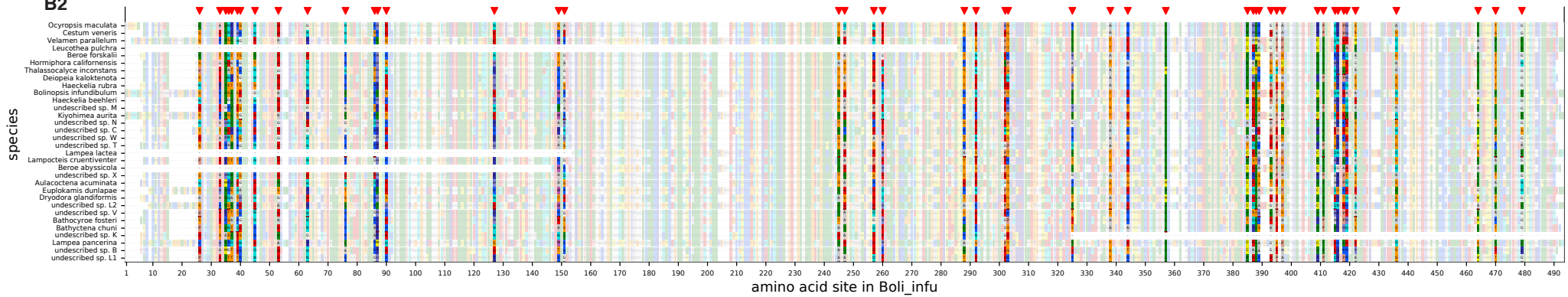

Supplement: icz063_Supplementary_Data [file icz063_supplementary_data.zip › icb-2019-0105-File013.pdf]
